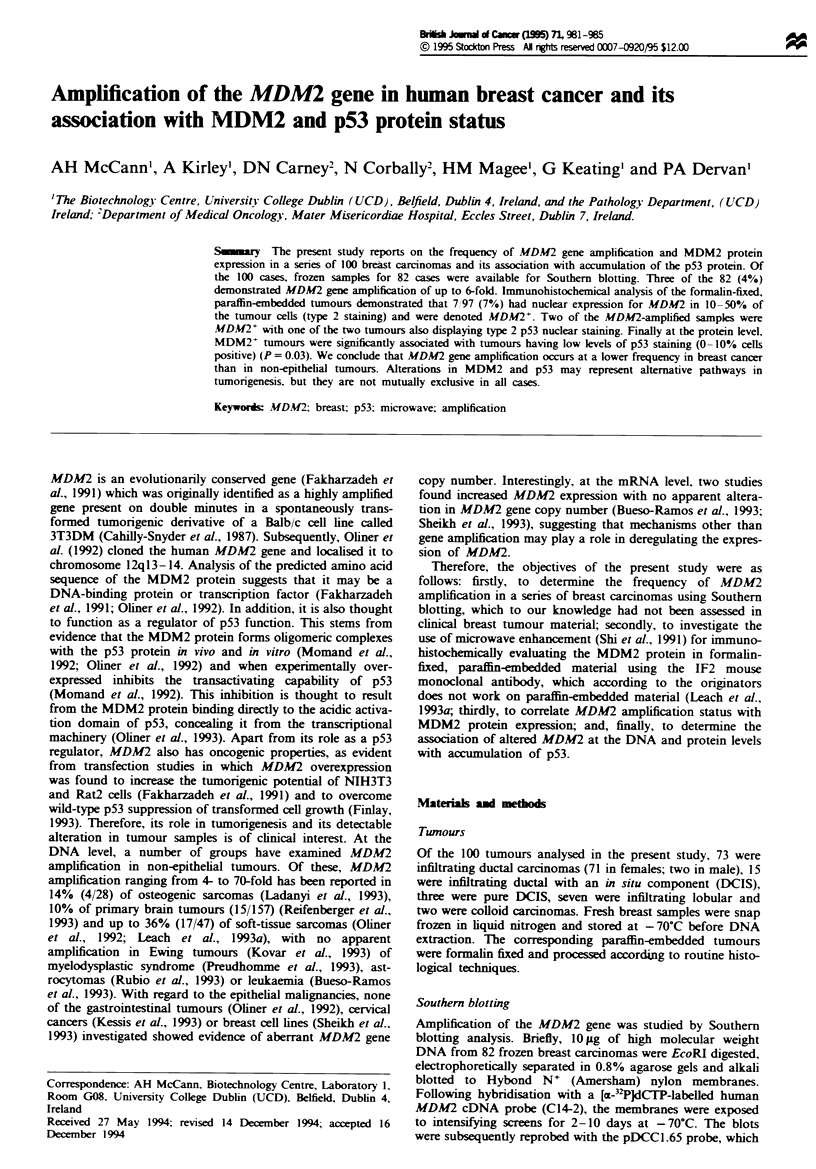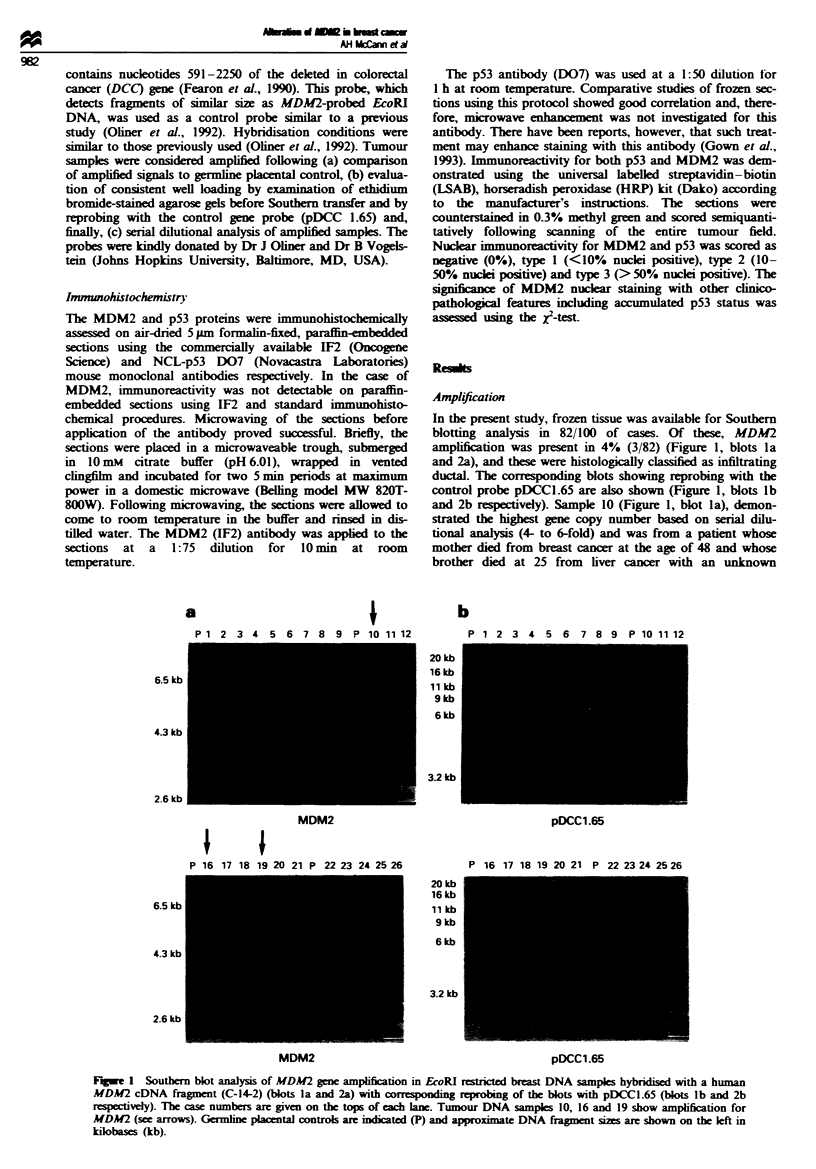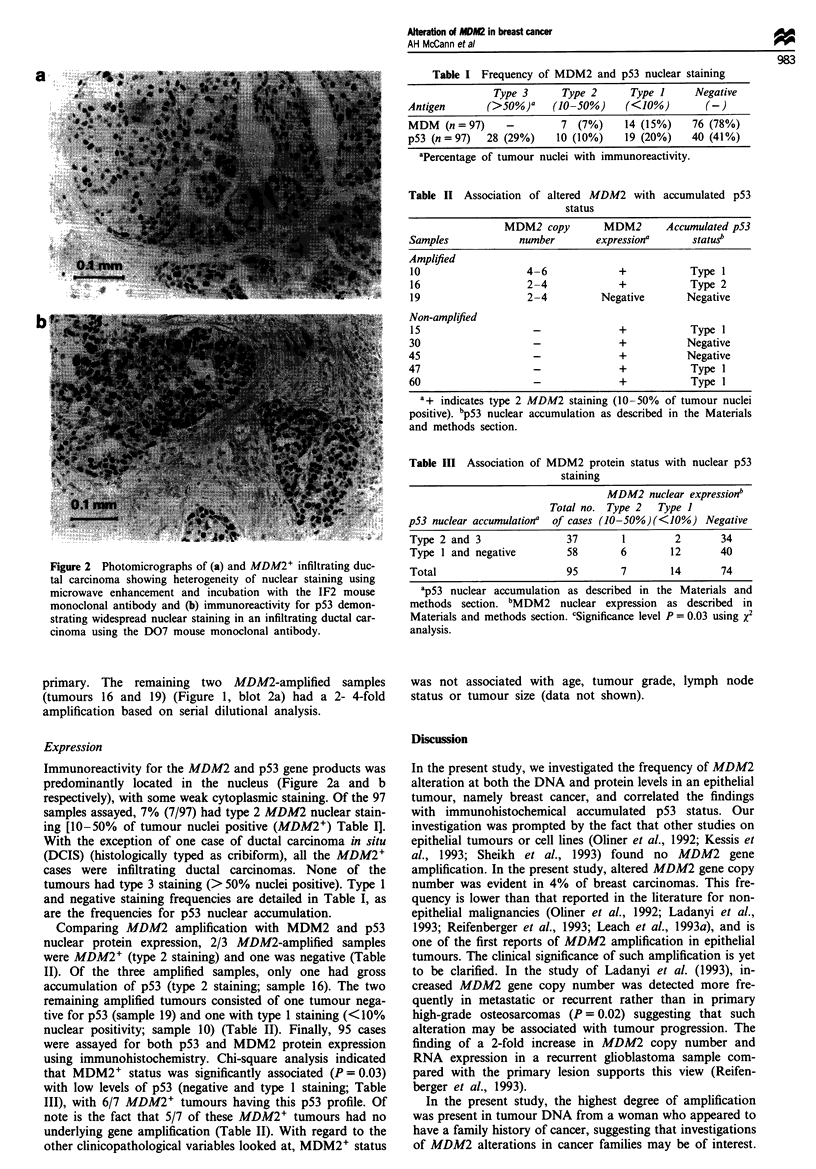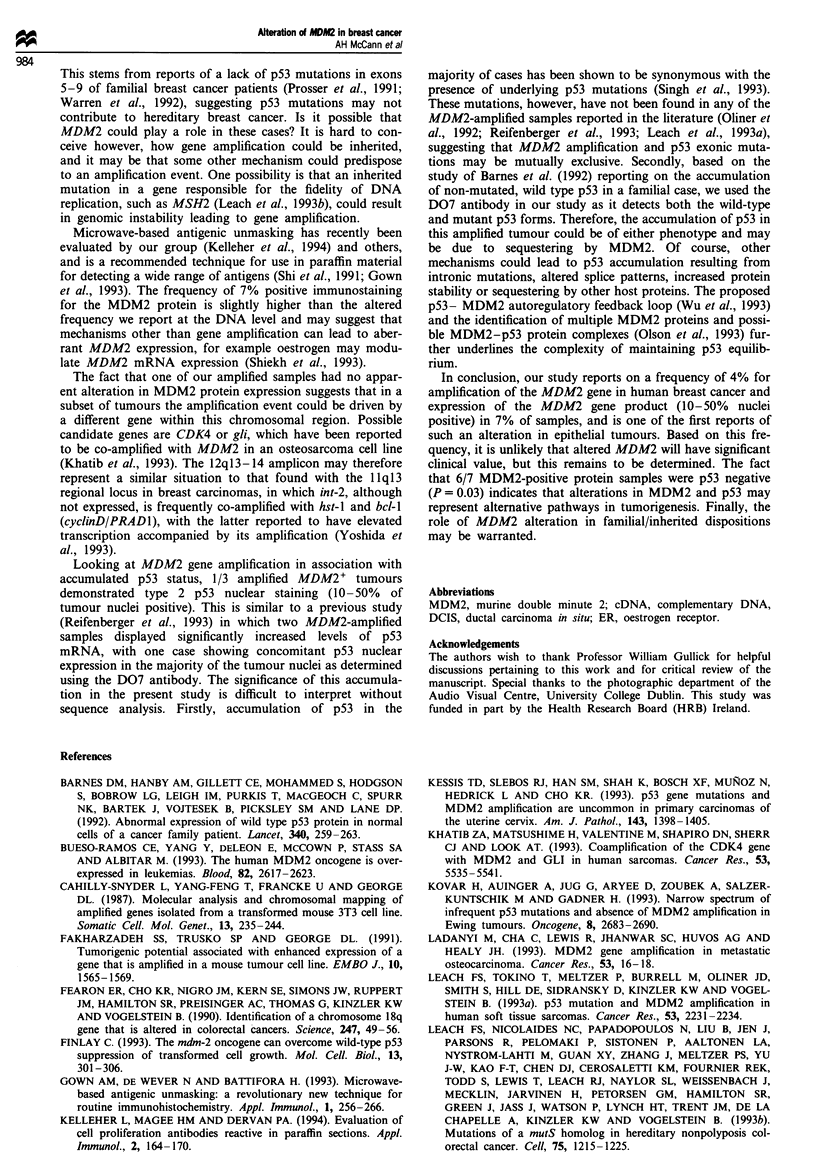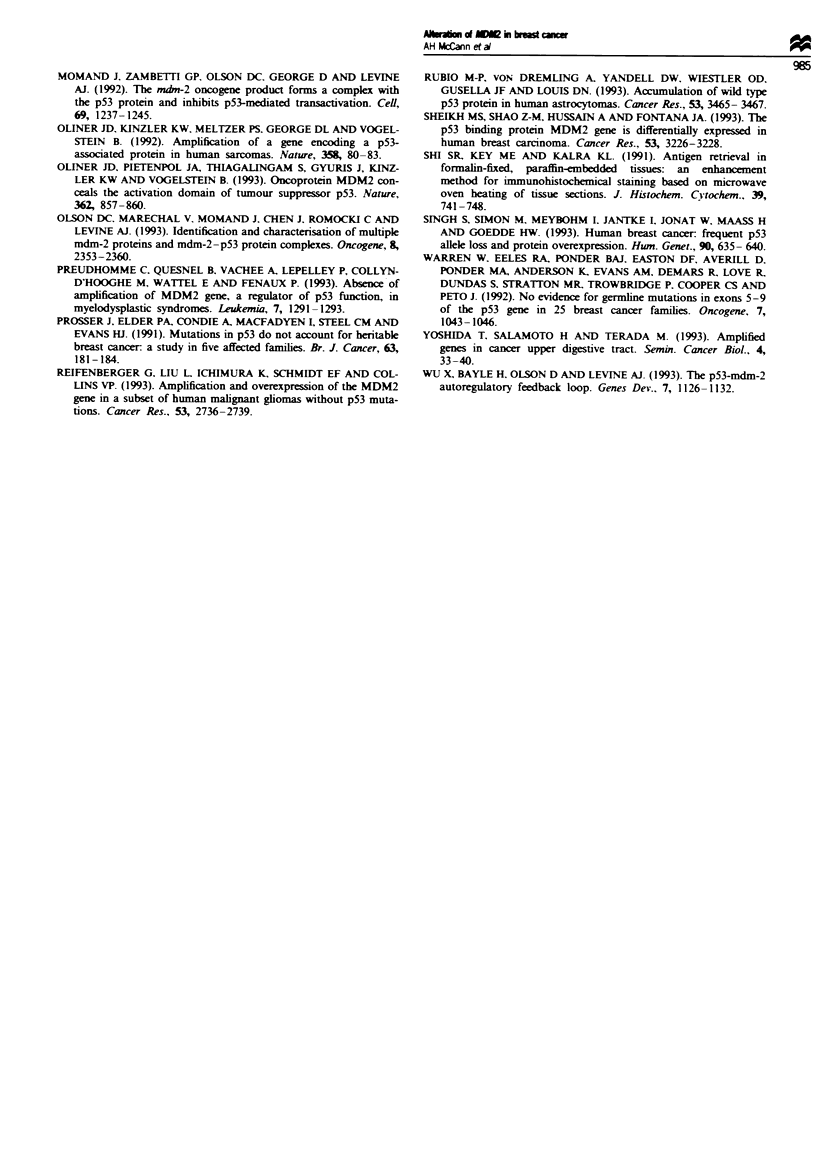# Amplification of the MDM2 gene in human breast cancer and its association with MDM2 and p53 protein status.

**DOI:** 10.1038/bjc.1995.189

**Published:** 1995-05

**Authors:** A. H. McCann, A. Kirley, D. N. Carney, N. Corbally, H. M. Magee, G. Keating, P. A. Dervan

**Affiliations:** Biotechnology Centre, University College Dublin, Belfield, Ireland.

## Abstract

**Images:**


British Journal of Cancer (1995) 71, 981-985

© 1995 Stockton Press All rights reserved 0007-0920/95 $12.00

### Amplification of the *MDM2* gene in human breast cancer and its association with MDM2 and p53 protein status

AH McCann[1], A Kirley[1], DN Carney[2], N Corbally[2], HM Magee[1], G Keating[1] and PA Dervan[1]

[1]*The Biotechnology Centre, University College Dublin (UCD), Belfield, Dublin 4, Ireland, and the Pathology Department, (UCD) Ireland;* [2]*Department of Medical Oncology, Mater Misericordiae Hospital, Eccles Street, Dublin 7, Ireland.*

**Summary** The present study reports on the frequency of *MDM2* gene amplification and MDM2 protein expression in a series of 100 breast carcinomas and its association with accumulation of the p53 protein. Of the 100 cases, frozen samples for 82 cases were available for Southern blotting. Three of the 82 (4%) demonstrated *MDM2* gene amplification of up to 6-fold. Immunohistochemical analysis of the formalin-fixed, paraffin-embedded tumours demonstrated that 7/97 (7%) had nuclear expression for *MDM2* in 10-50% of the tumour cells (type 2 staining) and were denoted *MDM2*$^+$. Two of the *MDM2*-amplified samples were *MDM2*$^+$ with one of the two tumours also displaying type 2 p53 nuclear staining. Finally at the protein level, MDM2$^+$ tumours were significantly associated with tumours having low levels of p53 staining (0-10% cells positive) ($P = 0.03$). We conclude that *MDM2* gene amplification occurs at a lower frequency in breast cancer than in non-epithelial tumours. Alterations in MDM2 and p53 may represent alternative pathways in tumorigenesis, but they are not mutually exclusive in all cases.

**Keywords:** *MDM2*; breast; p53; microwave; amplification

*MDM2* is an evolutionarily conserved gene (Fakharzadeh *et al.*, 1991) which was originally identified as a highly amplified gene present on double minutes in a spontaneously transformed tumorigenic derivative of a Balb/c cell line called 3T3DM (Cahilly-Snyder *et al.*, 1987). Subsequently, Oliner *et al.* (1992) cloned the human *MDM2* gene and localised it to chromosome 12q13-14. Analysis of the predicted amino acid sequence of the MDM2 protein suggests that it may be a DNA-binding protein or transcription factor (Fakharzadeh *et al.*, 1991; Oliner *et al.*, 1992). In addition, it is also thought to function as a regulator of p53 function. This stems from evidence that the MDM2 protein forms oligomeric complexes with the p53 protein *in vivo* and *in vitro* (Momand *et al.*, 1992; Oliner *et al.*, 1992) and when experimentally overexpressed inhibits the transactivating capability of p53 (Momand *et al.*, 1992). This inhibition is thought to result from the MDM2 protein binding directly to the acidic activation domain of p53, concealing it from the transcriptional machinery (Oliner *et al.*, 1993). Apart from its role as a p53 regulator, *MDM2* also has oncogenic properties, as evident from transfection studies in which *MDM2* overexpression was found to increase the tumorigenic potential of NIH3T3 and Rat2 cells (Fakharzadeh *et al.*, 1991) and to overcome wild-type p53 suppression of transformed cell growth (Finlay, 1993). Therefore, its role in tumorigenesis and its detectable alteration in tumour samples is of clinical interest. At the DNA level, a number of groups have examined *MDM2* amplification in non-epithelial tumours. Of these, *MDM2* amplification ranging from 4- to 70-fold has been reported in 14% (4/28) of osteogenic sarcomas (Ladanyi *et al.*, 1993), 10% of primary brain tumours (15/157) (Reifenberger *et al.*, 1993) and up to 36% (17/47) of soft-tissue sarcomas (Oliner *et al.*, 1992; Leach *et al.*, 1993*a*), with no apparent amplification in Ewing tumours (Kovar *et al.*, 1993) of myelodysplastic syndrome (Preudhomme *et al.*, 1993), astrocytomas (Rubio *et al.*, 1993) or leukaemia (Bueso-Ramos *et al.*, 1993). With regard to the epithelial malignancies, none of the gastrointestinal tumours (Oliner *et al.*, 1992), cervical cancers (Kessis *et al.*, 1993) or breast cell lines (Sheikh *et al.*, 1993) investigated showed evidence of aberrant *MDM2* gene copy number. Interestingly, at the mRNA level, two studies found increased *MDM2* expression with no apparent alteration in *MDM2* gene copy number (Bueso-Ramos *et al.*, 1993; Sheikh *et al.*, 1993), suggesting that mechanisms other than gene amplification may play a role in deregulating the expression of *MDM2*.

Therefore, the objectives of the present study were as follows: firstly, to determine the frequency of *MDM2* amplification in a series of breast carcinomas using Southern blotting, which to our knowledge had not been assessed in clinical breast tumour material; secondly, to investigate the use of microwave enhancement (Shi *et al.*, 1991) for immunohistochemically evaluating the MDM2 protein in formalin-fixed, paraffin-embedded material using the IF2 mouse monoclonal antibody, which according to the originators does not work on paraffin-embedded material (Leach *et al.*, 1993*a*; thirdly, to correlate *MDM2* amplification status with MDM2 protein expression; and, finally, to determine the association of altered *MDM2* at the DNA and protein levels with accumulation of p53.

### Materials and methods

### *Tumours*

Of the 100 tumours analysed in the present study, 73 were infiltrating ductal carcinomas (71 in females; two in male), 15 were infiltrating ductal with an *in situ* component (DCIS), three were pure DCIS, seven were infiltrating lobular and two were colloid carcinomas. Fresh breast samples were snap frozen in liquid nitrogen and stored at -70°C before DNA extraction. The corresponding paraffin-embedded tumours were formalin fixed and processed according to routine histological techniques.

### *Southern blotting*

Amplification of the *MDM2* gene was studied by Southern blotting analysis. Briefly, 10 µg of high molecular weight DNA from 82 frozen breast carcinomas were *Eco*RI digested, electrophoretically separated in 0.8% agarose gels and alkali blotted to Hybond N$^+$ (Amersham) nylon membranes. Following hybridisation with a [α-$^{32}$P]dCTP-labelled human *MDM2* cDNA probe (C14-2), the membranes were exposed to intensifying screens for 2-10 days at -70°C. The blots were subsequently reprobed with the pDCC1.65 probe, which

Correspondence: AH McCann, Biotechnology Centre, Laboratory 1, Room G08, University College Dublin (UCD), Belfield, Dublin 4, Ireland
Received 27 May 1994; revised 14 December 1994; accepted 16 December 1994

contains nucleotides 591–2250 of the deleted in colorectal cancer (*DCC*) gene (Fearon *et al.*, 1990). This probe, which detects fragments of similar size as *MDM2*-probed *Eco*RI DNA, was used as a control probe similar to a previous study (Oliner *et al.*, 1992). Hybridisation conditions were similar to those previously used (Oliner *et al.*, 1992). Tumour samples were considered amplified following (a) comparison of amplified signals to germline placental control, (b) evaluation of consistent well loading by examination of ethidium bromide-stained agarose gels before Southern transfer and by reprobing with the control gene probe (pDCC 1.65) and, finally, (c) serial dilutional analysis of amplified samples. The probes were kindly donated by Dr J Oliner and Dr B Vogelstein (Johns Hopkins University, Baltimore, MD, USA).

### *Immunohistochemistry*

The MDM2 and p53 proteins were immunohistochemically assessed on air-dried 5 μm formalin-fixed, paraffin-embedded sections using the commercially available IF2 (Oncogene Science) and NCL-p53 DO7 (Novacastra Laboratories) mouse monoclonal antibodies respectively. In the case of MDM2, immunoreactivity was not detectable on paraffin-embedded sections using IF2 and standard immunohistochemical procedures. Microwaving of the sections before application of the antibody proved successful. Briefly, the sections were placed in a microwaveable trough, submerged in 10 mM citrate buffer (pH 6.01), wrapped in vented clingfilm and incubated for two 5 min periods at maximum power in a domestic microwave (Belling model MW 820T-800W). Following microwaving, the sections were allowed to come to room temperature in the buffer and rinsed in distilled water. The MDM2 (IF2) antibody was applied to the sections at a 1:75 dilution for 10 min at room temperature.

The p53 antibody (DO7) was used at a 1:50 dilution for 1 h at room temperature. Comparative studies of frozen sections using this protocol showed good correlation and, therefore, microwave enhancement was not investigated for this antibody. There have been reports, however, that such treatment may enhance staining with this antibody (Gown *et al.*, 1993). Immunoreactivity for both p53 and MDM2 was demonstrated using the universal labelled streptavidin–biotin (LSAB), horseradish peroxidase (HRP) kit (Dako) according to the manufacturer's instructions. The sections were counterstained in 0.3% methyl green and scored semiquantitatively following scanning of the entire tumour field. Nuclear immunoreactivity for MDM2 and p53 was scored as negative (0%), type 1 (<10% nuclei positive), type 2 (10–50% nuclei positive) and type 3 (>50% nuclei positive). The significance of MDM2 nuclear staining with other clinicopathological features including accumulated p53 status was assessed using the $\chi^2$-test.

### Results

### *Amplification*

In the present study, frozen tissue was available for Southern blotting analysis in 82/100 of cases. Of these, *MDM2* amplification was present in 4% (3/82) (Figure 1, blots 1a and 2a), and these were histologically classified as infiltrating ductal. The corresponding blots showing reprobing with the control probe pDCC1.65 are also shown (Figure 1, blots 1b and 2b respectively). Sample 10 (Figure 1, blot 1a), demonstrated the highest gene copy number based on serial dilutional analysis (4- to 6-fold) and was from a patient whose mother died from breast cancer at the age of 48 and whose brother died at 25 from liver cancer with an unknown

**Figure 1** Southern blot analysis of *MDM2* gene amplification in *Eco*RI restricted breast DNA samples hybridised with a human *MDM2* cDNA fragment (C-14-2) (blots 1a and 2a) with corresponding reprobing of the blots with pDCC1.65 (blots 1b and 2b respectively). The case numbers are given on the tops of each lane. Tumour DNA samples 10, 16 and 19 show amplification for *MDM2* (see arrows). Germline placental controls are indicated (P) and approximate DNA fragment sizes are shown on the left in kilobases (kb).

**Figure 2** Photomicrographs of (**a**) and *MDM2*$^+$ infiltrating ductal carcinoma showing heterogeneity of nuclear staining using microwave enhancement and incubation with the IF2 mouse monoclonal antibody and (**b**) immunoreactivity for p53 demonstrating widespread nuclear staining in an infiltrating ductal carcinoma using the DO7 mouse monoclonal antibody.

**Table I** Frequency of MDM2 and p53 nuclear staining

| Antigen | *Type 3* (>50%)[a] | *Type 2* (10–50%) | *Type 1* (<10%) | *Negative* (–) |
|---|---|---|---|---|
| MDM ($n = 97$) | – | 7 (7%) | 14 (15%) | 76 (78%) |
| p53 ($n = 97$) | 28 (29%) | 10 (10%) | 19 (20%) | 40 (41%) |

[a]Percentage of tumour nuclei with immunoreactivity.

**Table II** Association of altered *MDM2* with accumulated p53 status

| *Samples* | MDM*2 copy number* | MDM*2 expression*[a] | *Accumulated p53 status*[b] |
|---|---|---|---|
| *Amplified* | | | |
| 10 | 4–6 | + | Type 1 |
| 16 | 2–4 | + | Type 2 |
| 19 | 2–4 | Negative | Negative |
| *Non-amplified* | | | |
| 15 | – | + | Type 1 |
| 30 | – | + | Negative |
| 45 | – | + | Negative |
| 47 | – | + | Type 1 |
| 60 | – | + | Type 1 |

[a]+ indicates type 2 *MDM2* staining (10–50% of tumour nuclei positive). [b]p53 nuclear accumulation as described in the Materials and methods section.

**Table III** Association of MDM2 protein status with nuclear p53 staining

| *p53 nuclear accumulation*[a] | *Total no. of cases* | *MDM2 nuclear expression*[b] *Type 2 (10–50%)* | *Type 1 (<10%)* | *Negative* |
|---|---|---|---|---|
| Type 2 and 3 | 37 | 1 | 2 | 34 |
| Type 1 and negative | 58 | 6 | 12 | 40 |
| Total | 95 | 7 | 14 | 74 |

[a]p53 nuclear accumulation as described in the Materials and methods section. [b]MDM2 nuclear expression as described in Materials and methods section. [c]Significance level $P = 0.03$ using $\chi^2$ analysis.

primary. The remaining two *MDM2*-amplified samples (tumours 16 and 19) (Figure 1, blot 2a) had a 2- 4-fold amplification based on serial dilutional analysis.

*Expression*

Immunoreactivity for the *MDM2* and p53 gene products was predominantly located in the nucleus (Figure 2a and b respectively), with some weak cytoplasmic staining. Of the 97 samples assayed, 7% (7/97) had type 2 *MDM2* nuclear staining [10–50% of tumour nuclei positive (*MDM2*$^+$) Table I]. With the exception of one case of ductal carcinoma *in situ* (DCIS) (histologically typed as cribiform), all the *MDM2*$^+$ cases were infiltrating ductal carcinomas. None of the tumours had type 3 staining (>50% nuclei positive). Type 1 and negative staining frequencies are detailed in Table I, as are the frequencies for p53 nuclear accumulation.

Comparing *MDM2* amplification with MDM2 and p53 nuclear protein expression, 2/3 *MDM2*-amplified samples were *MDM2*$^+$ (type 2 staining) and one was negative (Table II). Of the three amplified samples, only one had gross accumulation of p53 (type 2 staining; sample 16). The two remaining amplified tumours consisted of one tumour negative for p53 (sample 19) and one with type 1 staining (<10% nuclear positivity; sample 10) (Table II). Finally, 95 cases were assayed for both p53 and MDM2 protein expression using immunohistochemistry. Chi-square analysis indicated that MDM2$^+$ status was significantly associated ($P = 0.03$) with low levels of p53 (negative and type 1 staining; Table III), with 6/7 *MDM2*$^+$ tumours having this p53 profile. Of note is the fact that 5/7 of these *MDM2*$^+$ tumours had no underlying gene amplification (Table II). With regard to the other clinicopathological variables looked at, MDM2$^+$ status was not associated with age, tumour grade, lymph node status or tumour size (data not shown).

### Discussion

In the present study, we investigated the frequency of *MDM2* alteration at both the DNA and protein levels in an epithelial tumour, namely breast cancer, and correlated the findings with immunohistochemical accumulated p53 status. Our investigation was prompted by the fact that other studies on epithelial tumours or cell lines (Oliner *et al.*, 1992; Kessis *et al.*, 1993; Sheikh *et al.*, 1993) found no *MDM2* gene amplification. In the present study, altered *MDM2* gene copy number was evident in 4% of breast carcinomas. This frequency is lower than that reported in the literature for non-epithelial malignancies (Oliner *et al.*, 1992; Ladanyi *et al.*, 1993; Reifenberger *et al.*, 1993; Leach *et al.*, 1993*a*), and is one of the first reports of *MDM2* amplification in epithelial tumours. The clinical significance of such amplification is yet to be clarified. In the study of Ladanyi *et al.* (1993), increased *MDM2* gene copy number was detected more frequently in metastatic or recurrent rather than in primary high-grade osteosarcomas ($P = 0.02$) suggesting that such alteration may be associated with tumour progression. The finding of a 2-fold increase in *MDM2* copy number and RNA expression in a recurrent glioblastoma sample compared with the primary lesion supports this view (Reifenberger *et al.*, 1993).

In the present study, the highest degree of amplification was present in tumour DNA from a woman who appeared to have a family history of cancer, suggesting that investigations of *MDM2* alterations in cancer families may be of interest.

This stems from reports of a lack of p53 mutations in exons 5–9 of familial breast cancer patients (Prosser *et al.*, 1991; Warren *et al.*, 1992), suggesting p53 mutations may not contribute to hereditary breast cancer. Is it possible that *MDM2* could play a role in these cases? It is hard to conceive however, how gene amplification could be inherited, and it may be that some other mechanism could predispose to an amplification event. One possibility is that an inherited mutation in a gene responsible for the fidelity of DNA replication, such as *MSH2* (Leach *et al.*, 1993*b*), could result in genomic instability leading to gene amplification.

Microwave-based antigenic unmasking has recently been evaluated by our group (Kelleher *et al.*, 1994) and others, and is a recommended technique for use in paraffin material for detecting a wide range of antigens (Shi *et al.*, 1991; Gown *et al.*, 1993). The frequency of 7% positive immunostaining for the MDM2 protein is slightly higher than the altered frequency we report at the DNA level and may suggest that mechanisms other than gene amplification can lead to aberrant *MDM2* expression, for example oestrogen may modulate *MDM2* mRNA expression (Shiekh *et al.*, 1993).

The fact that one of our amplified samples had no apparent alteration in MDM2 protein expression suggests that in a subset of tumours the amplification event could be driven by a different gene within this chromosomal region. Possible candidate genes are *CDK4* or *gli*, which have been reported to be co-amplified with *MDM2* in an osteosarcoma cell line (Khatib *et al.*, 1993). The 12q13–14 amplicon may therefore represent a similar situation to that found with the 11q13 regional locus in breast carcinomas, in which *int-2*, although not expressed, is frequently co-amplified with *hst-1* and *bcl-1* (*cyclinD/PRAD1*), with the latter reported to have elevated transcription accompanied by its amplification (Yoshida *et al.*, 1993).

Looking at *MDM2* gene amplification in association with accumulated p53 status, 1/3 amplified $MDM2^+$ tumours demonstrated type 2 p53 nuclear staining (10–50% of tumour nuclei positive). This is similar to a previous study (Reifenberger *et al.*, 1993) in which two *MDM2*-amplified samples displayed significantly increased levels of p53 mRNA, with one case showing concomitant p53 nuclear expression in the majority of the tumour nuclei as determined using the DO7 antibody. The significance of this accumulation in the present study is difficult to interpret without sequence analysis. Firstly, accumulation of p53 in the majority of cases has been shown to be synonymous with the presence of underlying p53 mutations (Singh *et al.*, 1993). These mutations, however, have not been found in any of the *MDM2*-amplified samples reported in the literature (Oliner *et al.*, 1992; Reifenberger *et al.*, 1993; Leach *et al.*, 1993*a*), suggesting that *MDM2* amplification and p53 exonic mutations may be mutually exclusive. Secondly, based on the study of Barnes *et al.* (1992) reporting on the accumulation of non-mutated, wild type p53 in a familial case, we used the DO7 antibody in our study as it detects both the wild-type and mutant p53 forms. Therefore, the accumulation of p53 in this amplified tumour could be of either phenotype and may be due to sequestering by MDM2. Of course, other mechanisms could lead to p53 accumulation resulting from intronic mutations, altered splice patterns, increased protein stability or sequestering by other host proteins. The proposed p53- MDM2 autoregulatory feedback loop (Wu *et al.*, 1993) and the identification of multiple MDM2 proteins and possible MDM2–p53 protein complexes (Olson *et al.*, 1993) further underlines the complexity of maintaining p53 equilibrium.

In conclusion, our study reports on a frequency of 4% for amplification of the *MDM2* gene in human breast cancer and expression of the *MDM2* gene product (10–50% nuclei positive) in 7% of samples, and is one of the first reports of such an alteration in epithelial tumours. Based on this frequency, it is unlikely that altered *MDM2* will have significant clinical value, but this remains to be determined. The fact that 6/7 MDM2-positive protein samples were p53 negative ($P = 0.03$) indicates that alterations in MDM2 and p53 may represent alternative pathways in tumorigenesis. Finally, the role of *MDM2* alteration in familial/inherited dispositions may be warranted.

### Abbreviations

MDM2, murine double minute 2; cDNA, complementary DNA, DCIS, ductal carcinoma *in situ*; ER, oestrogen receptor.

### Acknowledgements

The authors wish to thank Professor William Gullick for helpful discussions pertaining to this work and for critical review of the manuscript. Special thanks to the photographic department of the Audio Visual Centre, University College Dublin. This study was funded in part by the Health Research Board (HRB) Ireland.

### References

BARNES DM, HANBY AM, GILLETT CE, MOHAMMED S, HODGSON S, BOBROW LG, LEIGH IM, PURKIS T, MACGEOCH C, SPURR NK, BARTEK J, VOJTESEK B, PICKSLEY SM AND LANE DP. (1992). Abnormal expression of wild type p53 protein in normal cells of a cancer family patient. *Lancet*, **340**, 259–263.

BUESO-RAMOS CE, YANG Y, DELEON E, MCCOWN P, STASS SA AND ALBITAR M. (1993). The human MDM2 oncogene is overexpressed in leukemias. *Blood*, **82**, 2617–2623.

CAHILLY-SNYDER L, YANG-FENG T, FRANCKE U AND GEORGE DL. (1987). Molecular analysis and chromosomal mapping of amplified genes isolated from a transformed mouse 3T3 cell line. *Somatic Cell. Mol. Genet.*, **13**, 235–244.

FAKHARZADEH SS, TRUSKO SP AND GEORGE DL. (1991). Tumorigenic potential associated with enhanced expression of a gene that is amplified in a mouse tumour cell line. *EMBO J.*, **10**, 1565–1569.

FEARON ER, CHO KR, NIGRO JM, KERN SE, SIMONS JW, RUPPERT JM, HAMILTON SR, PREISINGER AC, THOMAS G, KINZLER KW AND VOGELSTEIN B. (1990). Identification of a chromosome 18q gene that is altered in colorectal cancers. *Science*, **247**, 49–56.

FINLAY C. (1993). The *mdm*-2 oncogene can overcome wild-type p53 suppression of transformed cell growth. *Mol. Cell. Biol.*, **13**, 301–306.

GOWN AM, DE WEVER N AND BATTIFORA H. (1993). Microwave-based antigenic unmasking: a revolutionary new technique for routine immunohistochemistry. *Appl. Immunol.*, **1**, 256–266.

KELLEHER L, MAGEE HM AND DERVAN PA. (1994). Evaluation of cell proliferation antibodies reactive in paraffin sections. *Appl. Immunol.*, **2**, 164–170.

KESSIS TD, SLEBOS RJ, HAN SM, SHAH K, BOSCH XF, MUÑOZ N, HEDRICK L AND CHO KR. (1993). p53 gene mutations and MDM2 amplification are uncommon in primary carcinomas of the uterine cervix. *Am. J. Pathol.*, **143**, 1398–1405.

KHATIB ZA, MATSUSHIME H, VALENTINE M, SHAPIRO DN, SHERR CJ AND LOOK AT. (1993). Coamplification of the CDK4 gene with MDM2 and GLI in human sarcomas. *Cancer Res.*, **53**, 5535–5541.

KOVAR H, AUINGER A, JUG G, ARYEE D, ZOUBEK A, SALZER-KUNTSCHIK M AND GADNER H. (1993). Narrow spectrum of infrequent p53 mutations and absence of MDM2 amplification in Ewing tumours. *Oncogene*, **8**, 2683–2690.

LADANYI M, CHA C, LEWIS R, JHANWAR SC, HUVOS AG AND HEALY JH. (1993). MDM2 gene amplification in metastatic osteocarcinoma. *Cancer Res.*, **53**, 16–18.

LEACH FS, TOKINO T, MELTZER P, BURRELL M, OLINER JD, SMITH S, HILL DE, SIDRANSKY D, KINZLER KW AND VOGELSTEIN B. (1993*a*). p53 mutation and MDM2 amplification in human soft tissue sarcomas. *Cancer Res.*, **53**, 2231–2234.

LEACH FS, NICOLAIDES NC, PAPADOPOULOS N, LIU B, JEN J, PARSONS R, PELOMAKI P, SISTONEN P, AALTONEN LA, NYSTROM-LAHTI M, GUAN XY, ZHANG J, MELTZER PS, YU J-W, KAO F-T, CHEN DJ, CEROSALETTI KM, FOURNIER REK, TODD S, LEWIS T, LEACH RJ, NAYLOR SL, WEISSENBACH J, MECKLIN, JARVINEN H, PETORSEN GM, HAMILTON SR, GREEN J, JASS J, WATSON P, LYNCH HT, TRENT JM, DE LA CHAPELLE A, KINZLER KW AND VOGELSTEIN B. (1993*b*). Mutations of a *mutS* homolog in hereditary nonpolyposis colorectal cancer. *Cell*, **75**, 1215–1225.

MOMAND J, ZAMBETTI GP, OLSON DC, GEORGE D AND LEVINE AJ. (1992). The *mdm*-2 oncogene product forms a complex with the p53 protein and inhibits p53-mediated transactivation. *Cell*, **69**, 1237-1245.

OLINER JD, KINZLER KW, MELTZER PS, GEORGE DL AND VOGELSTEIN B. (1992). Amplification of a gene encoding a p53-associated protein in human sarcomas. *Nature*, **358**, 80-83.

OLINER JD, PIETENPOL JA, THIAGALINGAM S, GYURIS J, KINZLER KW AND VOGELSTEIN B. (1993). Oncoprotein MDM2 conceals the activation domain of tumour suppressor p53. *Nature*, **362**, 857-860.

OLSON DC, MARECHAL V, MOMAND J, CHEN J, ROMOCKI C AND LEVINE AJ. (1993). Identification and characterisation of multiple mdm-2 proteins and mdm-2-p53 protein complexes. *Oncogene*, **8**, 2353-2360.

PREUDHOMME C, QUESNEL B, VACHEE A, LEPELLEY P, COLLYN-D'HOOGHE M, WATTEL E AND FENAUX P. (1993). Absence of amplification of MDM2 gene, a regulator of p53 function, in myelodysplastic syndromes. *Leukemia*, **7**, 1291-1293.

PROSSER J, ELDER PA, CONDIE A, MACFADYEN I, STEEL CM AND EVANS HJ. (1991). Mutations in p53 do not account for heritable breast cancer: a study in five affected families. *Br. J. Cancer*, **63**, 181-184.

REIFENBERGER G, LIU L, ICHIMURA K, SCHMIDT EF AND COLLINS VP. (1993). Amplification and overexpression of the MDM2 gene in a subset of human malignant gliomas without p53 mutations. *Cancer Res.*, **53**, 2736-2739.

RUBIO M-P, VON DREMLING A, YANDELL DW, WIESTLER OD, GUSELLA JF AND LOUIS DN. (1993). Accumulation of wild type p53 protein in human astrocytomas. *Cancer Res.*, **53**, 3465-3467.

SHEIKH MS, SHAO Z-M, HUSSAIN A AND FONTANA JA. (1993). The p53 binding protein MDM2 gene is differentially expressed in human breast carcinoma. *Cancer Res.*, **53**, 3226-3228.

SHI SR, KEY ME AND KALRA KL. (1991). Antigen retrieval in formalin-fixed, paraffin-embedded tissues: an enhancement method for immunohistochemical staining based on microwave oven heating of tissue sections. *J. Histochem. Cytochem.*, **39**, 741-748.

SINGH S, SIMON M, MEYBOHM I, JANTKE I, JONAT W, MAASS H AND GOEDDE HW. (1993). Human breast cancer: frequent p53 allele loss and protein overexpression. *Hum. Genet.*, **90**, 635-640.

WARREN W, EELES RA, PONDER BAJ, EASTON DF, AVERILL D, PONDER MA, ANDERSON K, EVANS AM, DEMARS R, LOVE R, DUNDAS S, STRATTON MR, TROWBRIDGE P, COOPER CS AND PETO J. (1992). No evidence for germline mutations in exons 5-9 of the p53 gene in 25 breast cancer families. *Oncogene*, **7**, 1043-1046.

YOSHIDA T, SALAMOTO H AND TERADA M. (1993). Amplified genes in cancer upper digestive tract. *Semin. Cancer Biol.*, **4**, 33-40.

WU X, BAYLE H, OLSON D AND LEVINE AJ. (1993). The p53-mdm-2 autoregulatory feedback loop. *Genes Dev.*, **7**, 1126-1132.